# *N*-(2-Formyl­phen­yl)-4-meth­oxy-*N*-(4-meth­oxy­phenyl­sulfon­yl)benzene­sulfonamide

**DOI:** 10.1107/S1600536811047192

**Published:** 2011-11-12

**Authors:** Najat Abbassi, El Mostapha Rakib, Abdellah Hannioui, Hafid Zouihri

**Affiliations:** a Laboratoire de Chimie Organique et Analytique, Université Sultan Moulay Slimane, Faculté des Sciences et Techniques, Béni-Mellal, BP 523, Morocco; b Laboratoires de Diffraction des Rayons X, Centre Nationale pour la Recherche Scientifique et Technique, Rabat, Morocco

## Abstract

In the title compound, C_21_H_19_NO_7_S_2_, the dihedral angles between the formyl­phenyl ring and the two meth­oxy­phenyl rings are 33.87 (9) and 41.00 (10)°. The S atoms have a distorted tetra­hedral geometry and the N atom shows a trigonally planar [r.m.s. deviation = 0.0437 (13) Å] coordination. The crystal structure is stabilized by inter­molecular C—H⋯O hydrogen bonds.

## Related literature

For related structures, see: Abbassi *et al.* (2011*a* ▶,*b* ▶). For the biological activity of sulfonamides, see: Soledade *et al.* (2006 ▶); Lee & Lee (2002 ▶); Lopez *et al.* (2010 ▶); Zuercher *et al.* (2010 ▶). For the synthesis of 7-eth­oxy-*N*-alkyl­indazole derivatives, see: Abbassi *et al.* (2011*c* ▶).
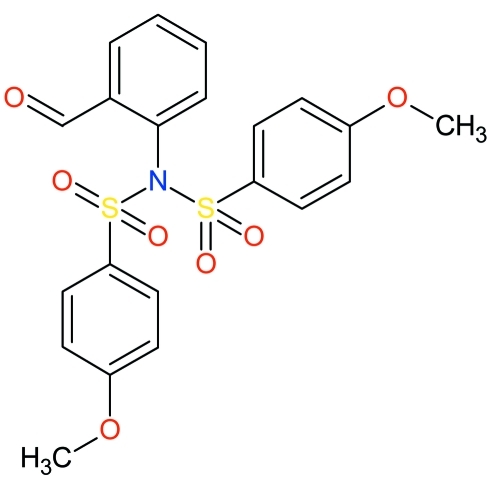


## Experimental

### 

#### Crystal data

C_21_H_19_NO_7_S_2_
*M* *_r_* = 461.49
Monoclinic, 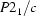

*a* = 9.0559 (3) Å
*b* = 25.8904 (10) Å
*c* = 9.3844 (3) Å
β = 103.423 (2)°
*V* = 2140.17 (13) Å^3^
*Z* = 4
Mo *K*α radiation
μ = 0.29 mm^−1^
*T* = 296 K
0.24 × 0.22 × 0.17 mm

#### Data collection

Bruker APEXII CCD detector diffractometer
37297 measured reflections
7971 independent reflections
4874 reflections with *I* > 2σ(*I*)
*R*_int_ = 0.034

#### Refinement

*R*[*F* ^2^ > 2σ(*F* ^2^)] = 0.047
*wR*(*F* ^2^) = 0.139
*S* = 1.01
7971 reflections
282 parameters
H-atom parameters constrained
Δρ_max_ = 0.40 e Å^−3^
Δρ_min_ = −0.35 e Å^−3^

### 

Data collection: *APEX2* (Bruker, 2005 ▶); cell refinement: *SAINT* (Bruker, 2005 ▶); data reduction: *SAINT*; program(s) used to solve structure: *SHELXS97* (Sheldrick, 2008 ▶); program(s) used to refine structure: *SHELXL97* (Sheldrick, 2008 ▶); molecular graphics: *PLATON* (Spek, 2009 ▶); software used to prepare material for publication: *publCIF* (Westrip, 2010 ▶).

## Supplementary Material

Crystal structure: contains datablock(s) I, global. DOI: 10.1107/S1600536811047192/bt5702sup1.cif

Structure factors: contains datablock(s) I. DOI: 10.1107/S1600536811047192/bt5702Isup2.hkl

Supplementary material file. DOI: 10.1107/S1600536811047192/bt5702Isup3.cml

Additional supplementary materials:  crystallographic information; 3D view; checkCIF report

## Figures and Tables

**Table 1:** Hydrogen-bond geometry (Å, °)

*D*—H⋯*A*	*D*—H	H⋯*A*	*D*⋯*A*	*D*—H⋯*A*
C14—H14⋯O4^i^	0.93	2.54	3.346 (2)	145
C16—H16⋯O2^ii^	0.93	2.45	3.237 (3)	143
C19—H19*B*⋯O6^iii^	0.96	2.59	3.455 (3)	151

Symmetry codes: (i) 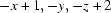; (ii) 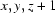; (iii) 
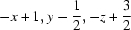
.

**Fig. 1.**
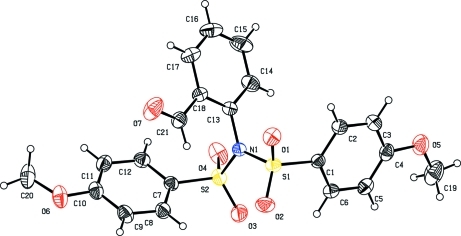
Molecular view of the title compound showing the atom-labeling scheme. Displacement ellipsoids are drawn at the 30% probability level. H atoms are represented as small spheres of arbitrary radii.

**Fig. 2.**
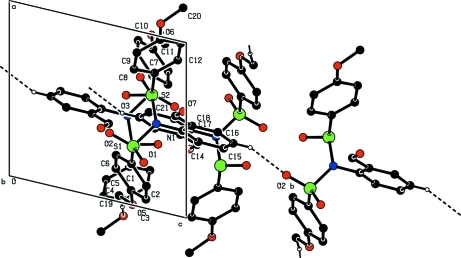
Partial packing view showing the C—H···O contacts as dashed lines. H atoms not involved in hydrogen bonds have been omitted for clarity.

## supplementary crystallographic information

### Comment

Sulfonamides constitute an important class of drugs (Lopez *et al.*, 2010; Zuercher *et al.*, 2010). They possess various types of pharmacological activities such as antibacterial, hypoglycemic, anti-inflammatory, and antitumor agents (Soledade *et al.*, 2006; Lee & Lee, 2002).

In former papers, we reported the crystal structures of *N*-(7-ethoxy-1*H*-indazol-4-yl)-4-methylbenzenesulfonamide (Abbassi *et al.*, 2011*a*) and *N*-[7-ethoxy-1-(prop-2-en-1-yl)-1*H*-indazol-4-yl]-4-methylbenzenesulfonamide (Abbassi *et al.*, 2011*b*). In this communication, the crystal structure of *N*-(2-formylphenyl)-4-methoxy-*N*-[(4-methoxyphenyl)sulfonyl]benzenesulfonamide is reported.

In the title compound, C_21_H_19_NO_7_S_2_, the C—S—N—S torsion angles are 83.22 (11)° and 110.03 (10)°, respectively. The dihedral angles between the two methoxyphenyl rings and the formylphenyl ring are 33.87 (9)° and 41.00 (10)°, respectively. The S atoms have a distorted tetrahedral geometry [maximum deviation: O—S—O = 119.93 (11)° and 120.36 (9)°, respectively].

In the crystal, molecules are connected by   intermolecular C—H···O hydrogen contacts.

### Experimental

A mixture of 2-nitrobenzaldehyde (1.22 mmol) and anhydrous SnCl_2_(1.1 g, 6.1 mmol) in 25 mL of absolute ethanol was stirred for 1 h. After reduction, the starting material disappeared, and the solution was allowed to cool down. The pH was made slightly basic (pH 7–8) by addition of 5% aqueous potassium bicarbonate before extraction with ethyl acetate. The organic phase was washed with brine and dried over magnesium sulfate. The solvent was removed to afford the amine, which was immediately dissolved in pyridine (5 ml) and then reacted with 4-methoxybenzenesulfonylchloride (0.26 g, 1.25 mmol) at room temperature for 24 h. After the reaction mixture was concentrated *in vacuo*, the resulting residue was purified by flash chromatography (eluted with Ethyl acetate: Hexane 3:7).

### Refinement

The H atoms were positioned geometrically and constrained to ride on their parent atoms with C—H = 0.93Å and *U*_iso_(H) = 1.2 *U*_eq_(C)  for CH, and C—H = 0.97 Å and *U*_iso_(H) = 1.5 *U*_eq_(C) for methyl groups. The methyl groups were allowed to rotate but not to tip.

### Crystal data

**Table:**

C_21_H_19_NO_7_S_2_	*F*(000) = 960
*M**_r_* = 461.49	*D*_x_ = 1.432 Mg m^−^^3^
Monoclinic, *P*2_1_/*c*	Mo *K*α radiation, λ = 0.71073 Å
Hall symbol: -P 2ybc	Cell parameters from 312 reflections
*a* = 9.0559 (3) Å	θ = 1.7–25.7°
*b* = 25.8904 (10) Å	µ = 0.29 mm^−^^1^
*c* = 9.3844 (3) Å	*T* = 296 K
β = 103.423 (2)°	Prism, colourless
*V* = 2140.17 (13) Å^3^	0.24 × 0.22 × 0.17 mm
*Z* = 4	

### Data collection

**Table:**

Bruker APEXII CCD detector diffractometer	4874 reflections with *I* > 2σ(*I*)
Radiation source: fine-focus sealed tube	*R*_int_ = 0.034
graphite	θ_max_ = 32.9°, θ_min_ = 2.3°
ω and φ scans	*h* = −12→13
37297 measured reflections	*k* = −39→39
7971 independent reflections	*l* = −14→14

### Refinement

**Table:**

Refinement on *F*^2^	Primary atom site location: structure-invariant direct methods
Least-squares matrix: full	Secondary atom site location: difference Fourier map
*R*[*F*^2^ > 2σ(*F*^2^)] = 0.047	Hydrogen site location: inferred from neighbouring sites
*wR*(*F*^2^) = 0.139	H-atom parameters constrained
*S* = 1.01	*w* = 1/[σ^2^(*F*_o_^2^) + (0.0614*P*)^2^ + 0.4769*P*] where *P* = (*F*_o_^2^ + 2*F*_c_^2^)/3
7971 reflections	(Δ/σ)_max_ < 0.001
282 parameters	Δρ_max_ = 0.40 e Å^−^^3^
0 restraints	Δρ_min_ = −0.35 e Å^−^^3^

### Special details

**Table:**

Geometry. All e.s.d.'s (except the e.s.d. in the dihedral angle between two l.s. planes) are estimated using the full covariance matrix. The cell e.s.d.'s are taken into account individually in the estimation of e.s.d.'s in distances, angles and torsion angles; correlations between e.s.d.'s in cell parameters are only used when they are defined by crystal symmetry. An approximate (isotropic) treatment of cell e.s.d.'s is used for estimating e.s.d.'s involving l.s. planes.
Refinement. Refinement of *F*^2^ against ALL reflections. The weighted *R*-factor *wR* and goodness of fit *S* are based on *F*^2^, conventional *R*-factors *R* are based on *F*, with *F* set to zero for negative *F*^2^. The threshold expression of *F*^2^ > σ(*F*^2^) is used only for calculating *R*-factors(gt) *etc*. and is not relevant to the choice of reflections for refinement. *R*-factors based on *F*^2^ are statistically about twice as large as those based on *F*, and *R*- factors based on ALL data will be even larger.

### Fractional atomic coordinates and isotropic or equivalent isotropic displacement parameters (Å^2^)

**Table:**

	*x*	*y*	*z*	*U*_iso_*/*U*_eq_	
C1	0.23459 (18)	0.05482 (6)	0.69043 (16)	0.0410 (3)	
C10	0.98208 (19)	0.20588 (7)	0.8526 (2)	0.0511 (4)	
C11	0.9883 (2)	0.17237 (8)	0.9684 (2)	0.0542 (4)	
C12	0.8887 (2)	0.13105 (7)	0.95227 (19)	0.0493 (4)	
C13	0.49327 (18)	0.11558 (6)	0.97998 (16)	0.0399 (3)	
C14	0.4509 (2)	0.07804 (8)	1.0687 (2)	0.0598 (5)	
C15	0.4479 (3)	0.09089 (11)	1.2108 (3)	0.0805 (7)	
C16	0.4896 (3)	0.13955 (11)	1.2651 (2)	0.0784 (7)	
C17	0.5319 (2)	0.17627 (9)	1.1779 (2)	0.0619 (5)	
C18	0.53257 (19)	0.16520 (6)	1.03307 (17)	0.0429 (3)	
C19	0.0364 (4)	−0.12570 (9)	0.6201 (3)	0.0954 (9)	
C2	0.1276 (2)	0.04938 (7)	0.7745 (2)	0.0504 (4)	
C20	1.1943 (3)	0.25456 (10)	0.9822 (3)	0.0869 (8)	
C21	0.5676 (2)	0.20678 (6)	0.9385 (2)	0.0495 (4)	
C3	0.0517 (2)	0.00361 (8)	0.7722 (2)	0.0571 (4)	
C4	0.0820 (2)	−0.03719 (7)	0.68792 (19)	0.0510 (4)	
C5	0.1864 (2)	−0.03151 (7)	0.6021 (2)	0.0534 (4)	
C6	0.2627 (2)	0.01458 (7)	0.60319 (19)	0.0493 (4)	
C7	0.78442 (18)	0.12355 (6)	0.82118 (18)	0.0421 (3)	
C8	0.7775 (2)	0.15749 (7)	0.7051 (2)	0.0523 (4)	
C9	0.8763 (2)	0.19839 (8)	0.7211 (2)	0.0571 (4)	
H11	1.0589	0.1775	1.0565	0.065*	
H12	0.8922	0.1084	1.0298	0.059*	
H14	0.4250	0.0449	1.0331	0.072*	
H15	0.4173	0.0664	1.2706	0.097*	
H16	0.4890	0.1474	1.3616	0.094*	
H17	0.5605	0.2089	1.2157	0.074*	
H19A	0.0129	−0.1187	0.5168	0.143*	
H19B	−0.0235	−0.1543	0.6393	0.143*	
H19C	0.1422	−0.1340	0.6525	0.143*	
H2	0.1078	0.0767	0.8317	0.060*	
H20A	1.1526	0.2629	1.0645	0.130*	
H20B	1.2588	0.2822	0.9652	0.130*	
H20C	1.2524	0.2233	1.0022	0.130*	
H21	0.5620	0.1994	0.8405	0.059*	
H3	−0.0205	−0.0001	0.8276	0.069*	
H5	0.2050	−0.0587	0.5439	0.064*	
H6	0.3328	0.0186	0.5456	0.059*	
H8	0.7065	0.1525	0.6172	0.063*	
H9	0.8723	0.2212	0.6438	0.068*	
N1	0.49649 (15)	0.10284 (5)	0.83139 (14)	0.0396 (3)	
O1	0.25834 (15)	0.15158 (5)	0.75974 (15)	0.0561 (3)	
O2	0.38046 (18)	0.12125 (6)	0.56618 (14)	0.0652 (4)	
O3	0.61869 (16)	0.05349 (6)	0.66024 (17)	0.0710 (4)	
O4	0.70366 (16)	0.03916 (5)	0.92655 (19)	0.0692 (4)	
O5	0.00311 (19)	−0.08133 (6)	0.69663 (17)	0.0741 (4)	
O6	1.07395 (16)	0.24749 (5)	0.85529 (19)	0.0709 (4)	
O7	0.6029 (2)	0.24957 (6)	0.98157 (19)	0.0891 (5)	
S1	0.33626 (5)	0.112283 (15)	0.69957 (4)	0.04289 (11)	
S2	0.65454 (5)	0.073091 (15)	0.80534 (5)	0.04803 (12)	

### Atomic displacement parameters (Å^2^)

**Table:**

	*U*^11^	*U*^22^	*U*^33^	*U*^12^	*U*^13^	*U*^23^
C1	0.0403 (8)	0.0443 (8)	0.0376 (7)	0.0003 (6)	0.0078 (6)	−0.0025 (6)
C10	0.0407 (9)	0.0420 (8)	0.0727 (12)	0.0003 (7)	0.0179 (8)	−0.0061 (8)
C11	0.0462 (9)	0.0587 (10)	0.0539 (10)	0.0005 (8)	0.0040 (8)	−0.0094 (8)
C12	0.0489 (9)	0.0522 (9)	0.0473 (9)	0.0040 (7)	0.0123 (7)	0.0042 (7)
C13	0.0432 (8)	0.0401 (7)	0.0380 (7)	0.0025 (6)	0.0125 (6)	0.0025 (6)
C14	0.0700 (13)	0.0521 (10)	0.0626 (12)	−0.0008 (9)	0.0264 (10)	0.0140 (8)
C15	0.0957 (18)	0.0942 (18)	0.0599 (13)	0.0078 (14)	0.0347 (12)	0.0329 (12)
C16	0.0986 (18)	0.1002 (18)	0.0400 (10)	0.0142 (15)	0.0232 (11)	0.0050 (11)
C17	0.0735 (13)	0.0689 (12)	0.0412 (9)	0.0103 (10)	0.0093 (9)	−0.0091 (8)
C18	0.0461 (9)	0.0443 (8)	0.0377 (8)	0.0041 (6)	0.0082 (6)	−0.0023 (6)
C19	0.121 (2)	0.0553 (13)	0.0980 (19)	−0.0222 (14)	0.0004 (17)	−0.0040 (13)
C2	0.0469 (9)	0.0545 (10)	0.0524 (9)	0.0013 (7)	0.0170 (7)	−0.0080 (7)
C20	0.0526 (13)	0.0722 (15)	0.131 (2)	−0.0136 (11)	0.0115 (13)	−0.0302 (14)
C21	0.0553 (10)	0.0393 (8)	0.0530 (9)	−0.0014 (7)	0.0106 (8)	−0.0028 (7)
C3	0.0516 (10)	0.0657 (11)	0.0570 (11)	−0.0075 (8)	0.0184 (8)	0.0001 (8)
C4	0.0508 (10)	0.0497 (9)	0.0463 (9)	−0.0068 (7)	−0.0017 (7)	0.0048 (7)
C5	0.0579 (11)	0.0492 (9)	0.0503 (10)	−0.0008 (8)	0.0069 (8)	−0.0101 (7)
C6	0.0511 (9)	0.0533 (9)	0.0451 (9)	−0.0009 (7)	0.0145 (7)	−0.0077 (7)
C7	0.0396 (8)	0.0424 (8)	0.0465 (8)	0.0006 (6)	0.0145 (6)	−0.0027 (6)
C8	0.0448 (9)	0.0634 (11)	0.0466 (9)	−0.0049 (8)	0.0063 (7)	0.0064 (8)
C9	0.0496 (10)	0.0588 (11)	0.0630 (11)	−0.0042 (8)	0.0134 (8)	0.0159 (9)
N1	0.0400 (7)	0.0402 (6)	0.0407 (7)	−0.0009 (5)	0.0135 (5)	−0.0059 (5)
O1	0.0575 (7)	0.0436 (6)	0.0652 (8)	0.0136 (5)	0.0105 (6)	−0.0017 (5)
O2	0.0867 (10)	0.0678 (9)	0.0444 (7)	−0.0073 (7)	0.0221 (7)	0.0097 (6)
O3	0.0611 (8)	0.0730 (9)	0.0863 (10)	−0.0102 (7)	0.0317 (7)	−0.0431 (8)
O4	0.0605 (8)	0.0425 (7)	0.1075 (12)	0.0113 (6)	0.0258 (8)	0.0203 (7)
O5	0.0837 (11)	0.0611 (9)	0.0737 (9)	−0.0243 (8)	0.0108 (8)	0.0015 (7)
O6	0.0547 (8)	0.0496 (7)	0.1075 (12)	−0.0100 (6)	0.0172 (8)	−0.0060 (7)
O7	0.1287 (15)	0.0457 (8)	0.0824 (11)	−0.0226 (9)	0.0034 (10)	−0.0064 (7)
S1	0.0497 (2)	0.0397 (2)	0.0399 (2)	0.00237 (16)	0.01165 (16)	0.00260 (14)
S2	0.0449 (2)	0.0373 (2)	0.0661 (3)	0.00037 (16)	0.02144 (19)	−0.00810 (17)

### Geometric parameters (Å, °)

**Table:**

C1—C2	1.391 (2)	C20—H20C	0.9600
C1—C6	1.385 (2)	C20—H20B	0.9600
C10—C9	1.389 (3)	C20—H20A	0.9600
C10—C11	1.381 (3)	C20—O6	1.427 (3)
C10—O6	1.358 (2)	C21—H21	0.9300
C11—H11	0.9300	C21—O7	1.197 (2)
C12—H12	0.9300	C3—H3	0.9300
C12—C11	1.385 (3)	C4—C5	1.385 (3)
C13—N1	1.4398 (19)	C4—C3	1.385 (3)
C13—C18	1.394 (2)	C4—O5	1.360 (2)
C13—C14	1.391 (2)	C5—H5	0.9300
C14—H14	0.9300	C6—H6	0.9300
C14—C15	1.380 (3)	C6—C5	1.378 (3)
C15—H15	0.9300	C7—C8	1.389 (2)
C15—C16	1.378 (4)	C7—C12	1.380 (2)
C16—H16	0.9300	C8—H8	0.9300
C17—H17	0.9300	C8—C9	1.371 (3)
C17—C16	1.366 (3)	C9—H9	0.9300
C18—C21	1.476 (2)	S1—C1	1.7412 (16)
C18—C17	1.390 (2)	S1—N1	1.6919 (14)
C19—H19C	0.9600	S1—O1	1.4272 (12)
C19—H19B	0.9600	S1—O2	1.4190 (13)
C19—H19A	0.9600	S2—C7	1.7411 (16)
C19—O5	1.424 (3)	S2—N1	1.6923 (13)
C2—H2	0.9300	S2—O4	1.4242 (15)
C2—C3	1.368 (3)	S2—O3	1.4184 (14)
			
C2—C1—S1	119.24 (13)	O6—C20—H20B	109.5
C6—C1—S1	120.27 (12)	O6—C20—H20A	109.5
C6—C1—C2	120.47 (16)	C18—C21—H21	118.3
C11—C10—C9	120.32 (16)	O7—C21—H21	118.3
O6—C10—C9	114.88 (17)	O7—C21—C18	123.38 (17)
O6—C10—C11	124.80 (18)	C4—C3—H3	119.8
C12—C11—H11	120.2	C2—C3—H3	119.8
C10—C11—H11	120.2	C2—C3—C4	120.30 (16)
C10—C11—C12	119.51 (17)	C3—C4—C5	120.25 (16)
C11—C12—H12	120.0	O5—C4—C5	124.27 (18)
C7—C12—H12	120.0	O5—C4—C3	115.49 (17)
C7—C12—C11	119.99 (16)	C4—C5—H5	120.1
C18—C13—N1	119.76 (13)	C6—C5—H5	120.1
C14—C13—N1	119.37 (15)	C6—C5—C4	119.77 (16)
C14—C13—C18	120.87 (16)	C1—C6—H6	120.2
C13—C14—H14	120.7	C5—C6—H6	120.2
C15—C14—H14	120.7	C5—C6—C1	119.69 (16)
C15—C14—C13	118.7 (2)	C8—C7—S2	120.07 (13)
C14—C15—H15	119.6	C12—C7—S2	119.40 (13)
C16—C15—H15	119.6	C12—C7—C8	120.44 (16)
C16—C15—C14	120.81 (19)	C7—C8—H8	120.2
C15—C16—H16	119.8	C9—C8—H8	120.2
C17—C16—H16	119.8	C9—C8—C7	119.53 (17)
C17—C16—C15	120.36 (19)	C10—C9—H9	119.9
C18—C17—H17	119.7	C8—C9—H9	119.9
C16—C17—H17	119.7	C8—C9—C10	120.20 (17)
C16—C17—C18	120.5 (2)	S1—N1—S2	124.80 (8)
C13—C18—C21	122.02 (14)	C13—N1—S2	116.88 (10)
C17—C18—C21	119.19 (16)	C13—N1—S1	117.92 (10)
C17—C18—C13	118.73 (16)	C4—O5—C19	118.16 (19)
H19B—C19—H19C	109.5	C10—O6—C20	117.59 (19)
H19A—C19—H19C	109.5	N1—S1—C1	105.45 (7)
O5—C19—H19C	109.5	O1—S1—C1	108.92 (8)
H19A—C19—H19B	109.5	O2—S1—C1	110.53 (8)
O5—C19—H19B	109.5	O1—S1—N1	103.45 (7)
O5—C19—H19A	109.5	O2—S1—N1	107.39 (8)
C1—C2—H2	120.3	O2—S1—O1	119.93 (8)
C3—C2—H2	120.3	N1—S2—C7	102.91 (7)
C3—C2—C1	119.50 (16)	O4—S2—C7	108.37 (9)
H20B—C20—H20C	109.5	O3—S2—C7	110.48 (9)
H20A—C20—H20C	109.5	O4—S2—N1	106.45 (8)
O6—C20—H20C	109.5	O3—S2—N1	106.78 (8)
H20A—C20—H20B	109.5	O3—S2—O4	120.37 (10)

### Hydrogen-bond geometry (Å, °)

**Table:**

*D*—H···*A*	*D*—H	H···*A*	*D*···*A*	*D*—H···*A*
C14—H14···O4^i^	0.93	2.54	3.346 (2)	145.
C16—H16···O2^ii^	0.93	2.45	3.237 (3)	143.
C19—H19B···O6^iii^	0.96	2.59	3.455 (3)	151.

Symmetry codes: (i) −*x*+1, −*y*, −*z*+2; (ii) *x*, *y*, *z*+1; (iii) −*x*+1, *y*−1/2, −*z*+3/2.
